# Protein-S-nitrosylation of adenovirus-5 E1A and human papillomavirus 16 E7 limits their ability to inhibit STING activity

**DOI:** 10.1128/jvi.01456-25

**Published:** 2025-10-13

**Authors:** Justin B. Cox, Eain A. Murphy

**Affiliations:** 1 Microbiology and Immunology Department, SUNY-Upstate Medical University 12302, Syracuse, New York, USA; Dartmouth College Geisel School of Medicine, Hanover, New Hampshire, USA

**Keywords:** HCMV, adenovirus, HPV, E1a, E7, nitrosylation, STING

## Abstract

All viruses that establish successful infections express proteins that inhibit innate anti-viral pathways such as the stimulator of interferon genes (STING) pathway. In response, cells have evolved mechanisms to limit viruses by modifying these viral proteins via post-translational modifications (PTMs). One potent PTM, protein-S-nitrosylation, inhibits the ability of human cytomegalovirus (HCMV) to undermine the establishment of an anti-viral state. The direct nitrosylation of HCMV tegument protein pp71 at a central cysteine within its pRB binding domain reduces pp71’s ability to limit the activity of STING. Two different proteins encoded by unrelated DNA viruses, adenovirus (AdV) E1A and human papillomavirus (HPV) E7, also contain a pRB binding domain and inhibit STING like pp71. Herein, we report that E1A and E7 are both protein-S-nitrosylated like pp71. Stable cell lines expressing a WT, or mutants in which the predicted modified cysteine was changed to the closely related serine amino acid, thus blocking protein-S-nitrosylation, revealed that E1A and E7 are both protein-S-nitrosylated. Furthermore, induction of the STING pathway promoted IFN-β1 transcript production and the phosphorylation of IRF3, which was limited in E1A and E7 stable cell lines. Mutant stable cell lines exhibited a stronger inhibition of IFN-β1 transcription and reduced IRF3 phosphorylation, suggesting that the PTM limits WT viral protein inhibition of STING. Furthermore, both E1a and E7 can complement the replication of a HCMV that lacks pp71 during times of STING activation. These observations support a model in which protein-S-nitrosylation of viral virulence factors may function as an anti-viral mechanism in DNA virus infections.

**IMPORTANCE:**

DNA viruses, such as HCMV, AdV, and HPV, have the capacity to cause significant disease. Infection with AdV can cause severe lower respiratory and liver disease in children, and HPV infection is persistent and is a causative agent of cancer. Thus, these infections can be a severe health risk. Host cells have adapted innate responses like protein S-nitrosylation to limit viral replication. Our previous work reported that direct nitrosylation of two HCMV viral proteins, pp65 and pp71, limits their ability to undermine host anti-viral responses. Herein, we investigated whether protein-S-nitrosylation of AdV and HPV proteins inhibits their functions, suggesting that this PTM is an anti-viral mechanism. This may provide insight into the development of broad anti-viral therapeutics for persistent viral infections.

## INTRODUCTION

Viral infections, such as those caused by common viruses like adenovirus (AdV) or human papillomavirus (HPV), are a significant burden to public health systems globally. AdV infections, although often self-resolving, can result in severe respiratory complications in children and adults with the capacity to cause significant disease and death in severe cases (1, 2). The pathogenesis induced by AdV is not restricted to the respiratory system, as it was recently reported that specific AdV strains can cause lethal infections in children, resulting in severe liver disease (3). A vaccine for AdV does exist, but it is not currently available to the general public and is provided only to those in the military (4). HPV is a common DNA virus that causes benign warts on the skin. However, specific genotypes of this virus can cause warts in the throat and uterus, which can develop into cancer (5–8). Although a vaccine is now available to the public, current guidelines suggest that vaccination should not be administered to individuals after 26 years of age, as one may have already been exposed to HPV. Thus, there is a need for the development of broadly neutralizing therapeutics for the control of persistent viral infections, such as AdV or HPV, that are opportunistic, targeting immune-naïve individuals.

The innate immunity of an infected host is the first line of defense for viral infections. Innate immune pathways are responsible for the recognition of molecular signals such as cytoplasmic double-stranded DNA (dsDNA) indicative of a viral intrusion. An important pathway in the recognition of cytoplasmic dsDNA is the stimulator of interferon genes (STING) pathway (9–12). The STING pathway is activated by cyclic guanosine adenosine synthase (cGAS) binding to dsDNA within the cytosol, which then covalently links ATP and GTP to form 2′3′-cGAMP, the activator of STING, resulting in the induction of interferon transcription (13, 14). Initiation of this pathway establishes a potent anti-viral response that inhibits the replication of multiple viral infections (15–17). However, viruses have evolved countermeasure mechanisms to limit the activity of this pathway early in infection, thus undermining the establishment of an anti-viral state within an infected cell. AdV-encoded E1A and HPV-encoded E7 directly inhibit STING by limiting its translocation to the cytosol, resulting in reduced activated TBK1 and a diminished interferon response (18, 19). Independent of this function, these proteins also inhibit the retinoblastoma protein (pRB), which is important for the G1 to S phase switch, thus inducing resting cells to initiate the cell cycle, thereby allowing for the replication of the viruses (20–24). E1A and E7 are both essential proteins for AdV and HPV, and in their absence, viral replication is severely attenuated (25–27).

Although E1A and E7 function to diminish interferon production within an infected host, the host has evolved multiple countermeasures to regulate the activity of these proteins. AdV E1A levels are regulated both transcriptionally and post-translationally, as a host protein, TRIM-35, ubiquitinates E1A at lysine K48, inducing its proteasomal degradation (28). Additionally, it was observed that in TRIM-35 overexpressing cells, there is a reduction in E1A transcription (28). E7 is regulated by PTMs that lead to reduced protein levels, including ubiquitination by Cullin-1 and Skp2 containing E3 ligase, which leads to its proteasomal degradation (29). Recently, Huang et al. reported that the activation of STING induces the phosphorylation of E7 by TBK1, leading to the degradation of the viral protein (30). PTMs like phosphorylation and ubiquitination regulate the function of viral proteins to establish an anti-viral state. However, there has been increasing evidence that an additional PTM, termed protein-S-nitrosylation, also functions as an anti-viral mechanism. In our previous work, we reported that protein-S-nitrosylation of human cytomegalovirus (HCMV) tegument proteins pp71 and pp65 serves as a potent anti-viral mechanism by blocking the biological functions of these key viral proteins (31, 32). pp71 is nitrosylated within its pRB binding domain of “LxCxE,” and when nitrosylation of this site is inhibited, the virus replicates with increased efficiency when compared with wild-type HCMV during conditions where STING is activated (32). Interestingly, pp71 has structural homology to both E1A and E7, in that they all possess this unique pRB binding domain (33). Coupled with the fact that each of these proteins is reported to inhibit STING (18, 34), we hypothesized that E1A and E7 are protein-S-nitrosylated like pp71 and protein-S-nitrosylation of E1A and E7 limits their ability to inhibit STING, thereby suggesting this PTM may function as a broad-based anti-viral mechanism.

Herein, we report that E7 and E1A are both protein-S-nitrosylated in the absence of infection. We also observed that simulation of the cGAS/STING pathway by 2′3′-cGAMP in E7 and E1A expressing cell lines results in a marked reduction in IFN-β1 transcripts and IRF3 phosphorylation, which is further inhibited in mutant E1A and E7 stable cell lines in which protein-S-nitrosylation is inhibited, suggesting that nitrosylation of these proteins limits their normal antagonizing abilities. Furthermore, E1A and E7 isoforms that cannot be protein-S-nitrosylated can both complement a pp71-deficient HCMV in the presence of activated STING. In sum, these data suggest that protein-S-nitrosylation may serve as a broad-based anti-viral mechanism for distinct viruses and thus provides a novel therapeutic target to limit viral replication.

## RESULTS

### AdV-5 E1A and HPV-16 E7 are protein-S-nitrosylated independent of viral infection

**Fig 1:**
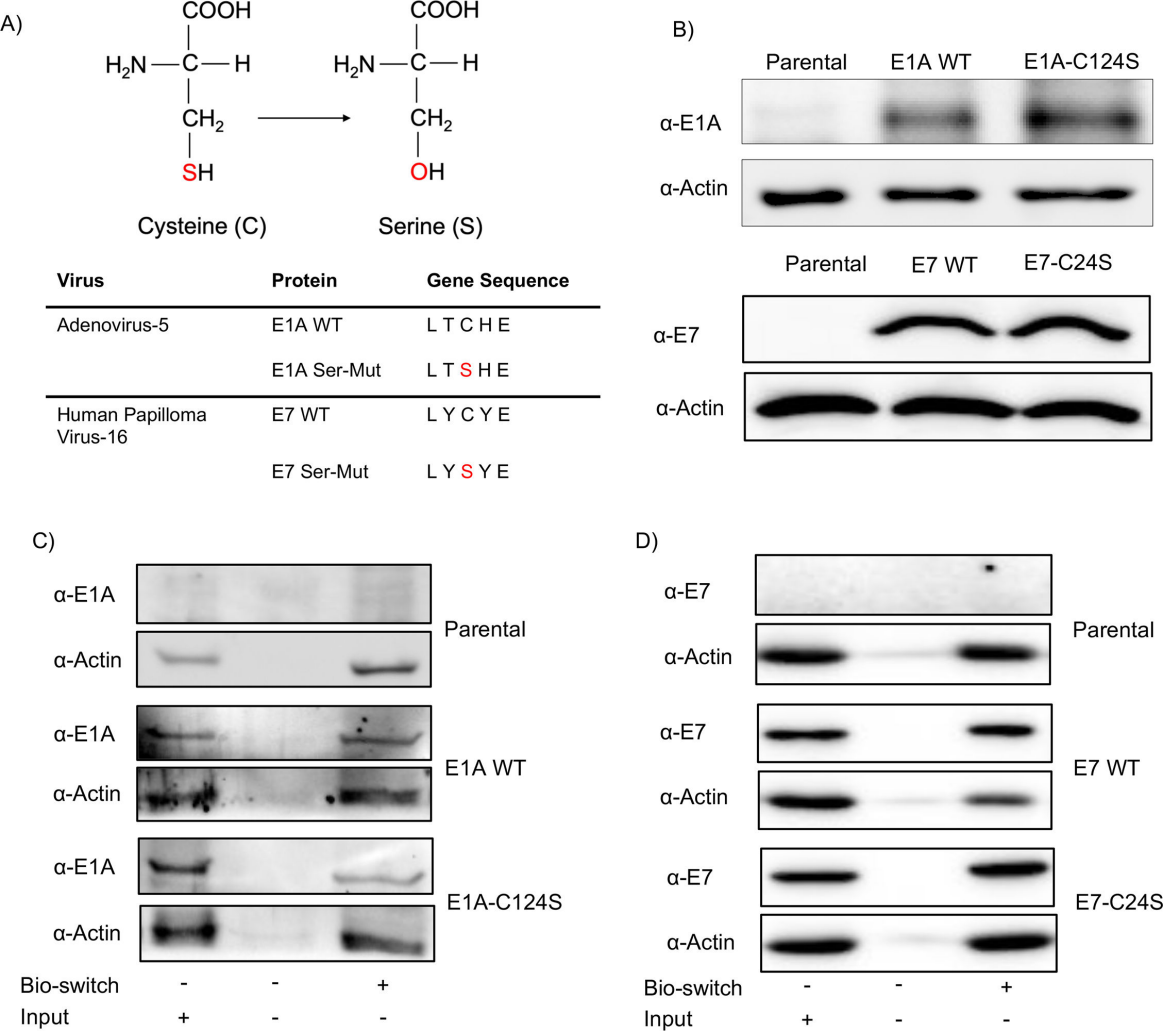
Adenovirus E1A and E7 are protein-S-nitrosylated. (**A**) The pRB binding domain of E1A and E7 WT and Ser-Mut sequences are shown. The red “S” indicates the serine amino acid mutation location. (**B**) NuFF-1 cells were transduced with lentivirus encoding WT and serine mutant E1A and E7 proteins. Protein expression was confirmed by western blotting. (**C, D**) Parental and WT or E1A-C124S and E7-C24S stable cell lines were subjected to a biotin switch. Protein lysates were then affinity purified by avidin purification, and the protein was analyzed by western blotting using the indicated antibodies. *n* = 3.

Stable fibroblast cell lines were generated by transduction of NuFF-1 cells with lentiviruses that express either WT or serine mutant isoforms of E1A and E7, termed E1A-C124S and E7-C24S. The central cysteine within the pRB binding domain of both viral proteins was mutated to the structurally related serine amino acid (Fig. 1A). Following drug selection, protein expression of E1A and E7 WT as well as the E1A-C124S and E7-C24S mutant isoforms was confirmed by western blotting. We observed similar amounts of protein expression within each of the E1A and E7 stable cell lines (Fig. 1B). To determine if E1A and E7 are protein-S-nitrosylated in NuFF-1 cells, cell lysates from E1A and E7 WT as well as the E1A-C124S and E7-C24S cells were subjected to a biotin switch assay followed by purification with avidin beads. The biotin switch assay changes all nitrosylated sites on a protein to biotin moieties. In the first part of the reaction, nitric oxide groups on cystines are unaltered, whereas the free thiol group on non-nitrosylated cystines is blocked by the addition of a chemical moiety. Following this, the nitric oxide groups on the protein-S-nitrosylated cystines are removed and replaced by biotin. Following the biotin switch and avidin purification, lysates were then immunoblotted using antibodies specific for E1A or E7. Actin is naturally nitrosylated at multiple sites and thus serves as a suitable loading control for western blotting after a biotin switch assay on cell lysates (35–37). We observed bands for WT E1A and E7 from the biotin-enriched lysates, suggesting that these proteins are S-nitrosylated (Fig. 1C and D). Furthermore, we observe slight bands in the mutant groups, but this is to be expected, as there is likely more than one cystine within these proteins that is modified by protein-S-nitrosylation in E1A or E7. The difference in band intensity in the western blots suggests that E7 is nitrosylated at multiple cysteine residues or may suggest that E7 may be endogenously biotinylated within the cell. It is highly probable that there are multiple protein-S-nitrosylated cystines in the proteins being studied (as we previously observed multiple protein-S-nitrosylation sites in both HCMV pp71 and pp65). To this point, E1a has 10 total cystines (including the pRB binding domain), and E7 has 5. Thus, we elected to only mutate the pRB binding domain cystines to serines as the role of this domain in STING inhibition has already been enumerated.

### IFN-β1 transcripts are reduced in AdV-5 E1A-C124S and HPV-16 E7-C24S stable cell lines

**Fig 2:**
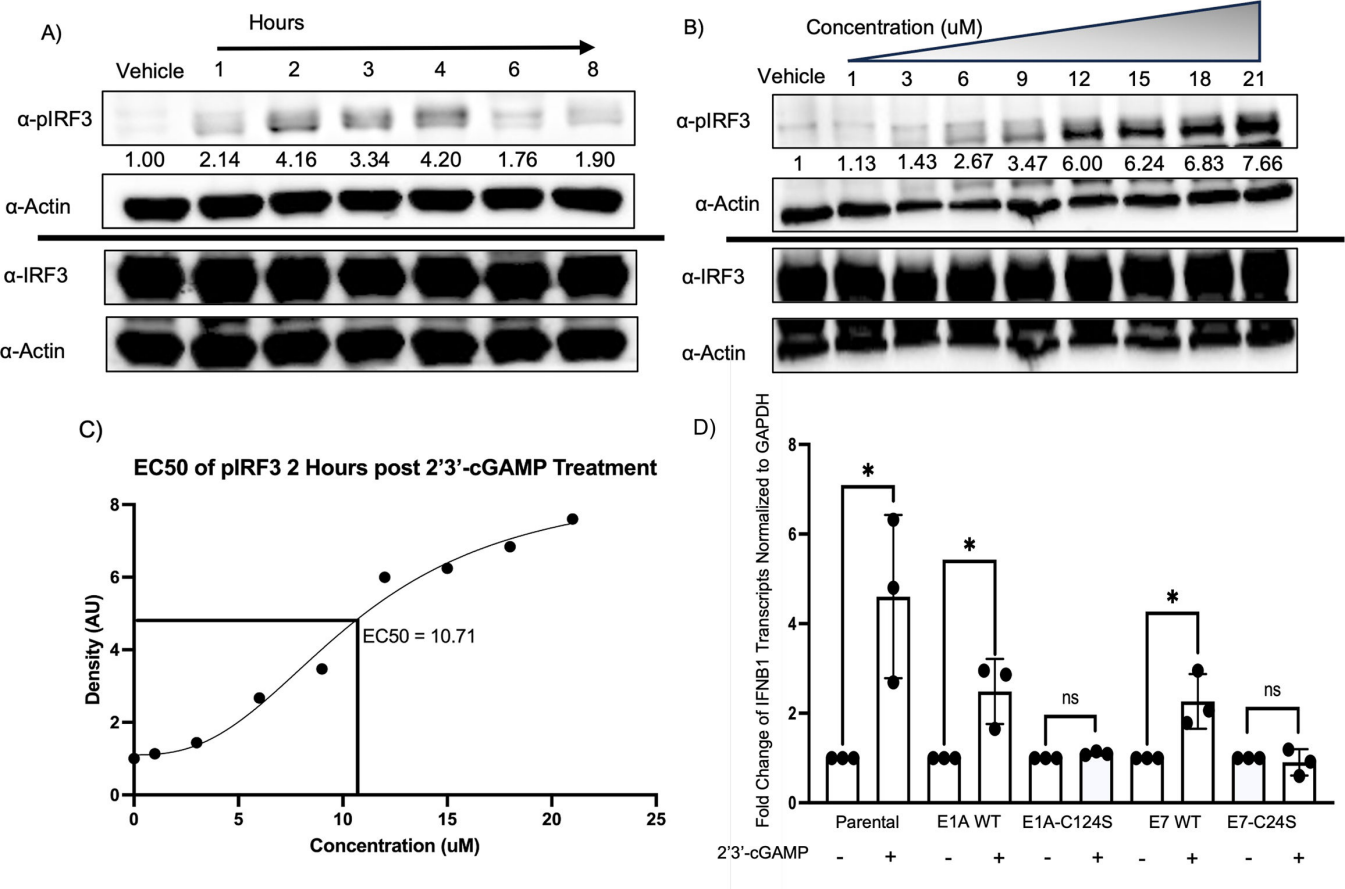
E1A and E7 serine mutant stable cell lines produce less interferon beta transcripts than parental cell lines. (**A**) NuFF-1 cells were treated with 20 μM of 2′3′-cGAMP, and protein was isolated at 1, 2, 3, 4, 6, and 8 h post-treatment. Protein was analyzed by western blotting, and densities were measured for band intensity and normalized to total IRF3. (**B**) NuFF-1 cells were treated with increasing concentrations of 2′3′-cGAMP, and protein was isolated 4 h post-treatment. Protein was analyzed by western blotting, and densities were measured for band intensity and normalized to total IRF3. (**C**) An EC50 analysis was performed on band intensities for all concentrations in panel **B**. (**D**) Parental NuFF-1, E1A, and E7 stable cell lines were treated with 10 μM of 2′3′-cGAMP for 24 h. RNA was then isolated and reverse-transcribed into cDNA and analyzed by qPCR for both IFNB1 and GAPDH. Fold change of ΔΔCt values, and all values are normalized to GAPDH. *n* = 3, *P* < 0.05*.

We next determined if interferon induction of WT and mutant stable cell lines demonstrates reduced STING activation compared with parental NuFF-1 cells following 2′3′-cGAMP treatment by quantifying the levels of IFN-β1 transcripts. To identify the optimal concentration of 2′3′-cGAMP treatment, fibroblasts were treated with 20 µM 2′3′-cGAMP, and protein lysates were collected over an 8 h time course post-treatment (Fig. 2A). Western blot analysis revealed optimal phosphorylation of IRF3 at 4 h post-transfection of 2′3′-cGAMP. To identify the optimal concentration of 2′3′-cGAMP, NuFF-1 fibroblasts were treated with increasing concentrations of the drug, and cell lysates were collected 4 h post-treatment. We observed a stepwise increase in the phosphorylation of IRF3, and EC50 analysis determined that the optimal concentration of 2′3′-cGAMP was 10 µM (Fig. 2B and C). These empirically determined time points and the concentration of 10 µM of 2′3′-cGAMP were used for all the subsequent experiments involving STING activation. Parental and stable cell lines expressing E1A and E7 were treated with 10 µM of 2′3′-cGAMP for 24 h, and then, RNA was isolated following treatment. As expected, parental cell lines produced IFN-β1 transcripts upon induction of the STING pathway. However, we observed a significant reduction in IFN-β1 transcript accumulation in WT expressing stable cell lines, and importantly, a further reduction of IFN-β1 transcripts for both E1A-C124S and E7-C24S (Fig. 2D). This suggests that E1A-C124S and E7-C24S cell lines that lack the capacity to be protein-S-nitrosylated within the pRB binding domain inhibit STING with better efficiency than their wild-type counterparts, thus blocking an anti-viral state.

### The phosphorylation of IRF3 in AdV-5 E1A-C124S and HPV-16 E7-C24S stable cell lines is inhibited

**Fig 3:**
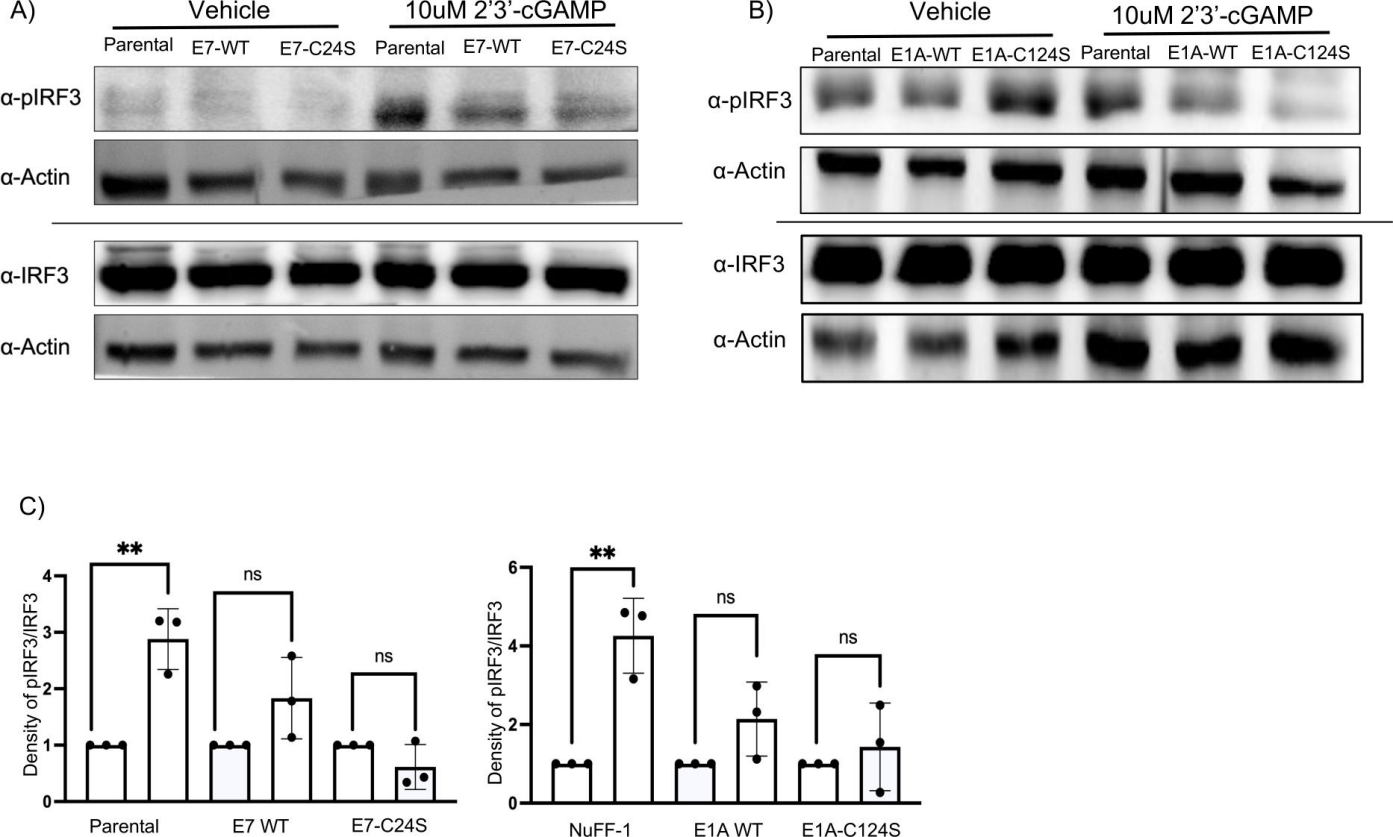
E1A-C124S and E7-C24S stable cell lines induce phospho-IRF3 levels less than WT E1A and E7 stable cell lines upon STING activation. (**A, B**) Stable cell lines were stimulated with 10 μM of 2′3′-cGAMP, and protein was isolated 4 h post-stimulation. Lysates were separated by SDS-PAGE, and protein levels were measured by western blotting with the indicated antibodies. (**C**) Densities for the western blots are shown for the quantification of IRF3 phosphorylation over total IRF3. *n* = 3, *P* < 0.01**.

Next, we wanted to determine the biological impact on the STING pathway of E1A and E7 when they cannot be nitrosylated at their pRB binding domain. To determine if stable cell lines expressing WT or E1A-C124S or E7-C24S induce phosphorylated IRF3 with different efficiencies upon STING activation, parental fibroblast cell lines and stable cell lines were treated with 10 µM of 2′3′-cGAMP for 4 h and then lysed to determine the phosphorylation levels of IRF3. As expected, parental cell lines had an increase in the phosphorylation status of IRF3 following treatment. As predicted, there was a decrease in the phosphorylation of IRF3 in WT E1A or E7 stable cell lines that were drug-treated when compared with non-transduced parental cell lines. Importantly, we observed a further reduction in the phosphorylation of IRF3 in the stable cell lines expressing the isoforms of E1A and E7 that cannot be protein-S-nitrosylated within their pRB-binding domains (Fig. 3A and B). Densitometry confirmed that WT and the serine mutant stable cell lines had a reduction in IRF3 phosphorylation (Fig. 3C). These data suggest that blocking nitrosylation of E1A and E7 provides the proteins with increased ability to antagonize STING in fibroblasts.

### AdV-5 E1A-C124S and HPV-16 E7-C24S stable cells replicate WT HCMV with higher efficiency during STING induction

**Fig 4:**
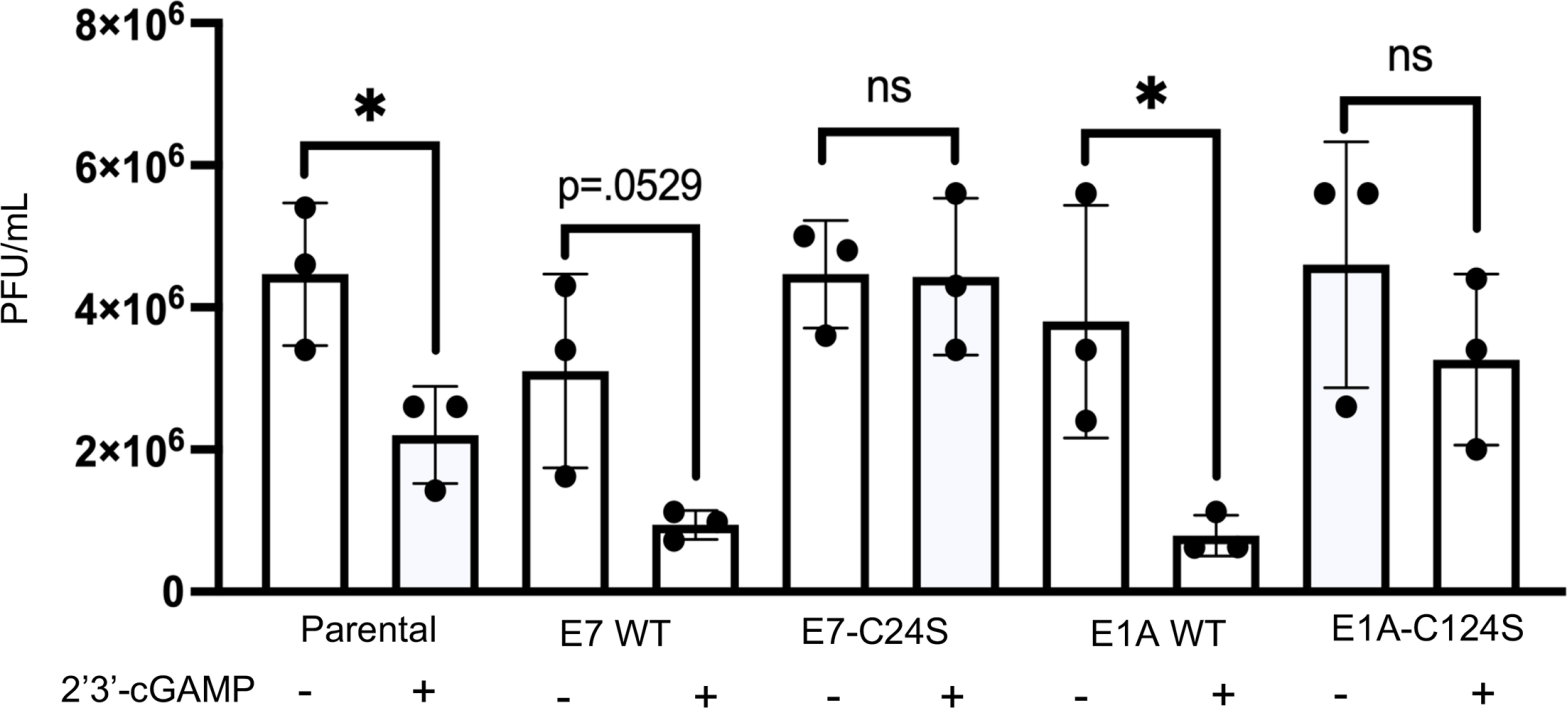
HCMV replication within E1A-C124S and E7-C24S cells is resistant to 2′3′-cGAMP treatment. Parental NuFF-1 cells and stable cell lines were mock-treated or pre-treated with 10 μM of 2′3′-cGAMP for 6 h. Following pre-treatment, all cells were infected with WT HCMV at an MOI of 1. Viral supernatants were collected 6 dpi, and then, cell-free virus production was quantified by TCid50 analysis on NuFF-1 cells. *n* = 3, *P* < 0.05*.

Our data thus far suggest that the nitrosylation of E1A and E7 impacts their ability to limit STING. Next, we tested if blocking nitrosylation of E1A and E7 had a biological impact on the replication of WT HCMV when the potent anti-viral STING pathway is induced. Parental fibroblasts, E1A or E7 stable cells, were treated with 10 μM of 2′3′-cGAMP, followed by infection at an MOI of 1 with WT HCMV. Viral supernatants were collected 6 days post-infection (dpi) and measured by TCid50. As expected, HCMV replicated in parental cell lines and all stable cell lines in the absence of 2′3′-cGAMP. We observed a significant decrease in the production of HCMV infectious virions in the 2′3′-cGAMP-treated parental NuFF-1 cells, indicating that 2′3′-cGAMP limits HCMV replication as expected. WT HCMV titers were also reduced in E1A and E7 WT stable cell lines, following 2′3′-cGAMP treatment. Importantly, we observed that E1A-C124S and E7-C24S WT HCMV titers were not reduced following 2′3′-cGAMP treatment (Fig. 4). These findings suggest that WT E1A and E7, which are liable to protein-S-nitrosylation by the host cell, are not resistant to the activation of STING, whereas nitrosylation-deficient E1A-C124S and E7-C24S can still allow for efficient viral replication after STING induction. This result supports a model in which blocking the nitrosylation of E1A and E7 allows the proteins to inhibit STING with better efficiency, thus restoring WT HCMV titers.

### AdV-5 E1A-C124S and HPV-16 E7-C24S stable cell lines complement the replication of HCMV that lacks expression of pp71 during STING induction

**Fig 5:**
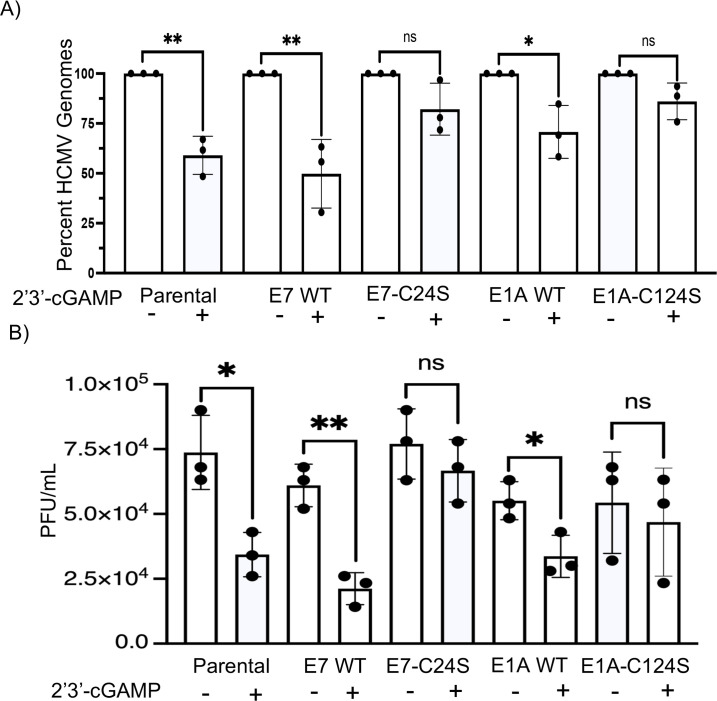
pp71-deficient HCMV replicates in serine mutant stable cell lines upon STING activation to similar levels seen in untreated cells. Parental NuFF-1 cells and stable cell lines were mock-treated or pre-treated with 10 μM of 2′3′-cGAMP for 6 h. Following pre-treatment, all cells were infected with Δpp71 HCMV at an MOI of 1. (**A**) DNA from infected cells was isolated 6 dpi, and viral genomes were quantitated with qPCR, measuring viral gene expression of UL123. All values were normalized to host genomes as measured with MDM2-specific primers. (**B**) Cell-associated virus was collected 6 dpi, and viral titers were measured by TCid50 on NuFF-1 cells. *n* = 3.

Our data suggest that E1A-C124S and E7-C24S allow WT HCMV to replicate after the induction of a pro-viral state. Next, we tested if E1A-C124S and E7-C24S complement the STING inhibition functions of pp71 by monitoring replication of a HCMV variant that lacks the capacity to express pp71 (32). Parental and stable cell lines were pre-treated with 10 µM of 2′3′-cGAMP and then infected with a pp71-deficient HCMV virus (Δpp71) at an MOI of 1. After 6 dpi, cell lysates were collected, and DNA was isolated from these cells to measure viral genomes by qPCR. We observed that HCMV genomes in parental NuFF-1 cells were reduced following 2′3′-cGAMP. The WT E1A and E7 stable cell lines were also reduced following 2′3′-cGAMP treatment. The percentage of HCMV genomes in all untreated groups was similar. Importantly, the genomes of Δpp71 in infection of E1A-C124S and E7-C24S stable cell lines were not significantly reduced after inducing STING (Fig. 5A). This suggests that E1A-C124S and E7-C24S stable cell lines are resistant to 2′3′-cGAMP treatment and may complement pp71’s function in the inhibition of STING, at least in viral DNA replication. To determine if this blocking of the STING pathway results in complementation of Δpp71 HCMV infectious virus production, parental, E1A, and E7 stable cell lines were pre-treated with 10 µM of 2′3′-cGAMP for 6 h and then infected with Δpp71 HCMV. Cell-associated virus was collected at 6 dpi and then quantified by TCid50. Cell-associated virus was collected to ensure accurate monitoring of viral replication, as HCMV typically spreads cell to cell. Untreated parental and WT E1A and E7 stable cell lines replicated Δpp71 HCMV to similar levels. However, following 2′3′-cGAMP treatment, the parental, E1A, and E7 WT stable cell lines demonstrated a significant reduction in HCMV titers, suggesting that E1A and E7 WT stable cell lines, which are still suitable substrates for protein-S-nitrosylation, are still limited by the induction of STING. Importantly, there was no significant reduction of Δpp71 HCMV titers following 2′3′-cGAMP treatment in E1A-C124S and E7-C24S stable cell lines, suggesting that they allowed Δpp71 HCMV replication to levels similar to their untreated counterparts (Fig. 5B). These data suggest that both E1A-C124S and E7-C24S can complement pp71’s ability to inhibit STING. In sum, we observed diminished STING induction of an anti-viral response in E1A-C124S and E7-C24S, suggesting that blocking nitrosylation of E1A and E7 allows the proteins to antagonize STING with better efficiency. These findings also highlight that E1A-C124S and E7-C24S can complement pp71’s ability to limit STING activity as a Δpp71 HCMV replicated to equal levels in treated and untreated stable cell lines.

## DISCUSSION

Our previous work identified multiple HCMV proteins that were post-translationally modified by protein-S-nitrosylation and characterized a novel mechanism of host regulation in which protein-S-nitrosylation limits the pro-viral functions of pp65 and pp71 during HCMV infection of fibroblasts (31, 32). This led us to question whether protein-S-nitrosylation of viral proteins is an anti-viral mechanism in DNA viruses that impacts virulence factors. HCMV pp71 contains a consensus pRB binding domain, LxCxE/D, that is found in two other proteins critical for efficient viral replication, E1A and E7 (18, 33). These domains are highly similar—the only known protein S-nitrosylation motif identified in GAPDH, I/LxCxxE/D (38). Furthermore, each of these proteins, in the context of their respective infections, inhibits the activity of STING. This convergence led us to hypothesize that E1A and/or E7 may be protein-S-nitrosylated in a similar fashion as pp71, and this protein modification would impact their biological functions. To this end, stable cell lines expressing WT E1A or E7 revealed that both proteins are protein-S-nitrosylated (Fig. 1C and D). This PTM did not require viral infection, suggesting that to a certain level, fibroblasts induce the modification in the absence of a stress-induced event. It should be noted that the complete protein-S-nitrosylation status of E1A and E7 remains unknown. Future mass spectrometry analysis of the two proteins may reveal multiple nitrosylation sites on E1A and E7, as we observed multiple sites on pp71 to be S-nitrosylated (32), and our biotin switch assay suggests that there is more than one nitrosylation site on the proteins (Fig. 1C and D). It may be the case that blocking all sites on E1A or E7 further exaggerates their antagonizing ability of STING. However, we did not observe this effect in pp71, as blocking nitrosylation at the pRB binding domain was sufficient in improving its ability to antagonize STING (32). We did not have the capacity to test whether altering the central cystine to the closely related serine within the pRB binding domain of E1A and E7 impacts their ability to interact with pRB. It remains an attractive possibility that protein-S-nitrosylation is also a regulator of this biological function of these two proteins.

Protein-S-nitrosylation of E1A and E7 resulted in a biological outcome that suggests this PTM is inhibitory to the viral proteins. Once STING is activated, it will translocate from the rough endoplasmic reticulum to the Golgi, where it interacts with TBK1 (11, 39). This ultimately leads to the activation of transcription factors like IRF3, leading to interferon and inhibitory cytokine production (10, 11). Secreted IFN-β1 can then stimulate both the infected cell and adjacent cells, resulting in an anti-viral state through IRF9 and STAT1 activation (40, 41). In fact, in our model system, IFN-β1 transcripts were induced in fibroblasts following 2′3′-cGAMP treatment as expected. However, the levels of IFN-β1 were induced to lower levels in stable cell lines that expressed the viral proteins. Importantly, we observed a significantly lower induction of IFN-β1 transcripts in the serine mutant stable cell lines (Fig. 2), indicating that these proteins may antagonize STING with better efficiency in support of a model in which protein-S-nitrosylation of viral proteins is inhibitory to their functions.

Following 2′3′-cGAMP treatment in parental cells, we observed a strong increase in the phosphorylation status of IRF3, which was less pronounced in the WT E1A and E7 stable cells, as these proteins are reported to limit STING induction. E1A-C124S and E7-C24S stable cells had further reduced levels of the phosphorylation of IRF3 compared with WT stable cell lines (Fig. 3A and B). Importantly, there was little difference in total IRF3 protein levels, suggesting that the reduction in phosphorylation of IRF3 is from the reduced activity of STING in the serine mutant cells and not a reduction in total IRF3. This observed reduction in phosphorylation of IRF3 suggests that both E1A and E7, in the absence of additional viral proteins, are sufficient for inhibiting the STING pathway. Importantly, variants of these proteins that are resistant to protein-S-nitrosylation within their pRB binding domains demonstrated a more potent inhibition of the STING pathway. Based on these findings, a consistent model emerges in which protein-S-nitrosylation supplements the host’s anti-viral response.

In our model system, STING is antagonized with higher efficiency in cells that express E1A and E7 isoforms that are limited in their ability to be protein-S-nitrosylated. This next led us to determine if this translates to an advantage for the replicating virus. In the absence of 2′3′-cGAMP, we observed that WT E1A and E7 stable cell lines produced infectious virions similar to levels found in parental NuFF-1 cells. These titers were reduced in parental and WT E1A and E7 cells following STING induction. However, in E1A-C214S and E7-C24S cell lines, we observed that HCMV viral titers were not reduced following 2′3′-cGAMP treatment (Fig. 4). This suggests that blocking nitrosylation of E1A and E7 at their pRB-binding domain provides the proteins an advantage to antagonize STING. These findings support our model that nitrosylation can limit E1A and E7’s ability to limit STING activity. It is possible that using infectious HPV and AdV infection may yield different results, but our current results show that expressions of E1A-C124S and E7-C24S alone provide a distinct advantage to the replication of an unrelated virus, HCMV, in an activated anti-viral state.

HCMV tegument protein pp71 possesses several functional similarities to E1A and E7. E1A and E7 both inhibit STING and contain a pRB binding domain similar in structure to pp71 (18, 42). To determine if E1A and E7 can functionally complement the lack of pp71 during HCMV infection, NuFF-1 cells were infected with a Δpp71 HCMV. Δpp71 HCMV has a growth defect in the infection of NuFF-1 cells due to its inability to activate viral IE gene expression (42). Parental and E1A and E7 WT expressing NuFF-1 cells infected with Δpp71 HCMV in the absence of STING induction replicated viral genomes to similar levels, and as expected, genomes for the parental cells and stable cell lines were reduced following 2′3′-cGAMP treatment. However, the genomes in the E1A-C124S and E7-C24S stable cell lines were not reduced following 2′3′-cGAMP treatment, suggesting that they can diminish the effects of STING activation (Fig. 5A). Following 2′3′-cGAMP treatment, Δpp71 HCMV titers were similar in the E1A-C124S and E7-C24S stable cell lines compared with their untreated counterparts (Fig. 5B). These data suggest that E1A and E7, which are still liable to protein-S-nitrosylation, are not able to fully restore the biological functions of pp71, but E1A-C124S and E7-C24S stable cell lines still complement pp71’s ability to limit STING. Our findings highlight that blocking protein-S-nitrosylation of E1A and E7 effectively attenuates the proteins’ ability to limit STING activity. These results support a model in which blocking nitrosylation of E1A and E7 provides the proteins an advantage in antagonizing STING, but these proteins cannot fully complement pp71 in HCMV infection, as the wild-type E1A and E7 expressing cell lines still resulted in a significant growth defect of Δpp71 HCMV when compared with WT HCMV (Fig. 4 and 5). Our data suggest that the key biological functions of pp71 for HCMV infection may be required to support HCMV replication. HCMV pp71 is a multifunctional protein involved in the inhibition of hDAXX, pRB, and STING and stimulates the MIEP in HCMV infection (34, 42–45) and thus is a critical tegument protein in HCMV infection. We believe that E7 and E1A do not have the capacity to compensate for all the functions of HCMV infection, resulting in the same replication kinetics in parental cell lines. There is evidence that HPV E7 can complement the function of HCMV UL97, but no other evidence suggests that it can compensate for the loss of pp71, further highlighting the importance of our findings that E7-C24S can complement pp71’s ability to limit STING (46). In sum, our data suggest that E7 and E1A do not fully complement all pp71’s functions but still antagonize STING induction in the absence of pp71. We observe that serine mutant E1A and E7 are resistant to STING activation, highlighting that protein-S-nitrosylation is an anti-viral mechanism in multiple DNA virus infections.

**Fig 6:**
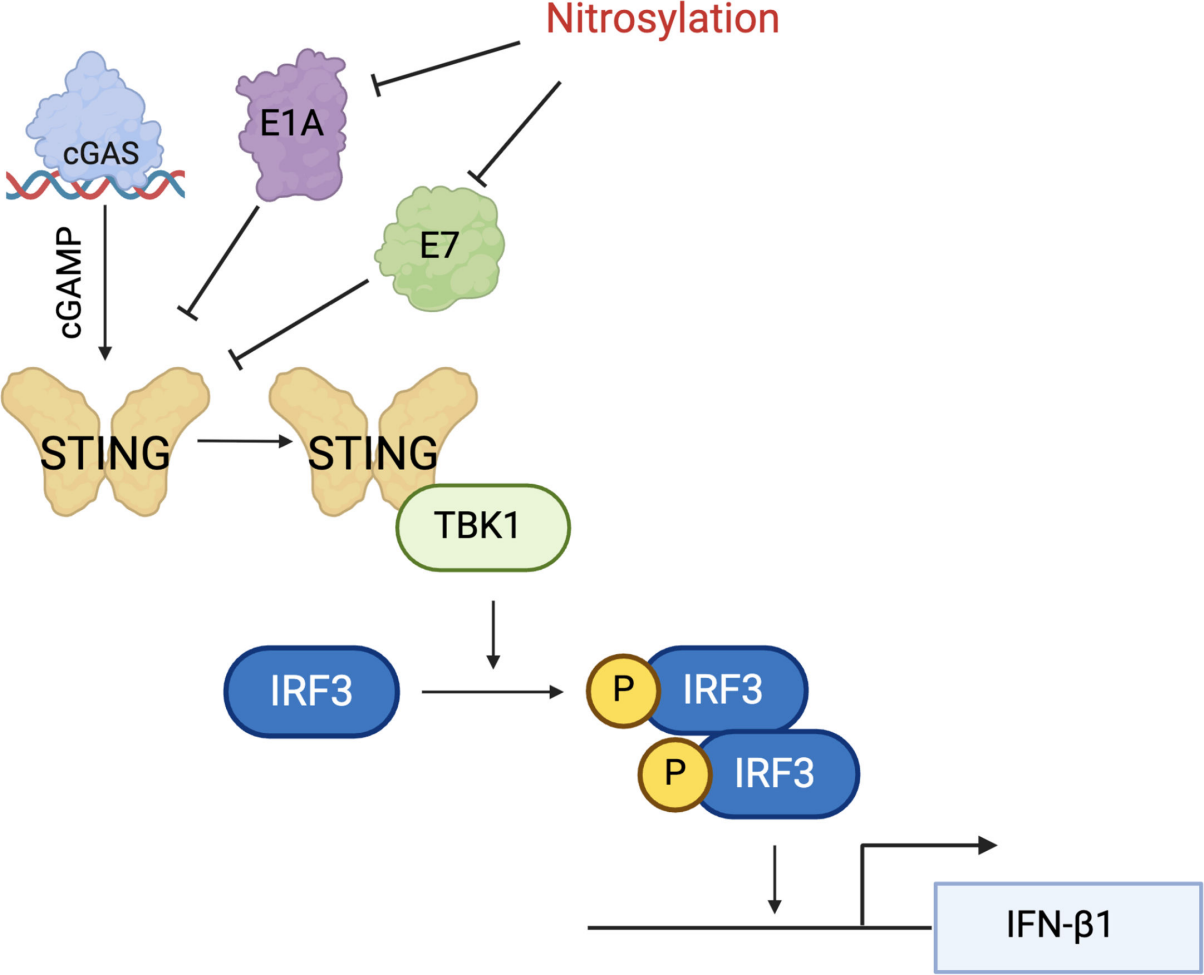
Protein-S-nitrosylation of E1A and E7 is an antiviral mechanism. NuFF-1 cells expressing E1A and E7 are inhibited in their antagonism of STING function by protein-S-nitrosylation. Cells expressing an E1A-C124S or E7-C24S phosphorylate IRF3 with lower efficiency, resulting in less INFB1 transcription compared with WT E1A and E7 and a diminished antiviral state.

In sum, our work identifies two additional proteins that are regulated by protein-S-nitrosylation, suggesting that this modification functions as an anti-viral mechanism. The identification of protein-S-nitrosylation of E1A and E7 represents a novel discovery and may provide evidence for exploiting applications in medicine for increasing protein-S-nitrosylation. By blocking protein-S-nitrosylation of E1A and E7 at their pRB-binding domain, we identified that both proteins antagonize STING with higher efficiency, leading to a reduction in IFN-β1 transcripts. Our data further suggest that this may be from the lack of phosphorylation of IRF3 in serine mutant stable cell lines, allowing the proteins to antagonize STING with higher efficiency (Fig. 6). Importantly, we observed that E1A-C124S and E7-C24S cell lines are resistant to 2′3′-cGAMP stimulation in WT and Δpp71 HCMV infection. Identifying additional factors regulated by protein-S-nitrosylation suggests that this may be a conserved and broadly neutralizing anti-viral mechanism that has evolved to limit infection by distinct viral family members. This is supported by the recent report that inhibition of nitric oxide, a key substrate for protein-S-nitrosylation, allows for higher HCMV titers and that treatment with nitric oxide donors attenuates HCMV replication, in support of our model (47). This modification may be exploited therapeutically to control drug-resistant or -persistent viral infections. Identifying additional viral factors that are protein-S-nitrosylated will further provide evidence that protein-S-nitrosylation regulates multiple viral infections and has broadly neutralizing properties.

## MATERIALS AND METHODS

### Cell culture

Newborn fetal fibroblasts (NuFF-1) and HEK-293T cells were maintained in Dulbecco’s modified essential medium (Cleveland Clinic) supplemented with 10% fetal bovine serum (FBS) (Millipore-Sigma), 1% penicillin-streptomycin solution (Cleveland Clinic), and 2 mM L-Glutamine (Cleveland Clinic). Cells were maintained at 5% carbon dioxide at 37°C. Cells infected with HCMV for the expansion of the virus were supplemented with complete medium that contains 10% newborn calf serum (Gibco). All cells were split with 0.5% Trypsin/EDTA (Cleveland Clinic) at a dilution of 1:2, and fibroblast passages were recorded to limit cellular senescence and limited to no more than 30 passages for experiments.

### Virus propagation

HCMV genomes were contained within bacterial artificial chromosomes (BAC) in SW105 bacteria. For the isolation of HCMV BACs, bacteria were streaked onto Luria Broth (LB) agar-chloramphenicol plates and grown at 32°C for 24 h. The bacteria were then cultured overnight in LB-chloramphenicol broth, and the next day, BAC DNA was isolated and transfected with electroporation into MRC5 cells that were at 50% confluency (32). The next day, the medium was changed and incubated until the plate demonstrated 100% cytopathic effect (CPE). The virus was then isolated by collecting the supernatant and cells, sonicating, and centrifuging the virus at 72,000 *× g* for 1.5 h at 18°C in a 20% sorbitol cushion with an SW-28 rotor in a Beckman-Coulter ultracentrifuge. Virus was titered on fibroblasts by TCid50 to measure PFU/mL.

### Lentiviral production and transduction

HEK-293T cells were seeded at 80% confluency 1 day before transfection. To form DNA:Lipofectamine complexes, 5.6 μg of E1A-WT (VB230516-1368xug), E1A-C124S (VB230516-1367tkb), E7-WT (VB240412-1392fuy), and E7-C24S (VB240413-1083mrr) pLV-eGFP-T2A-Puro (Vector Builder), 7.1 μg of p-CMV-VSV-G, and 14.2 μg of pDR 8.91 were incubated with 60 μL of Lipofectamine 2000 (Thermo-Fisher) in Opti-MEM for 30 min at room temperature. Protein expression of the viral proteins is driven by a CMV promoter that is contained between the lentivirus LTRs. DNA:Lipofectamine complexes were overlayed onto HEK-293T cells overnight, and the following day, media were changed to 10% NCS supplemented with 2 mM L-Glutamine. The supernatant was collected at 48 and 72 h post-medijm change, filtered with a 0.45 μm filter (Sigma), and the media were overlayed on NuFF-1 cells at 70% confluency. NuFF-1 cells were then selected with 700 ng/mL of puromycin (Invitrogen) and allowed to grow for two doublings. Protein expression was confirmed with western blotting.

### Measuring viral titers

Human cytomegalovirus was titered by the TCid50 assay. NuFF-1 cells were seeded to 90% confluency in a 96-well plate 24 h prior to infection. Viral supernatant was serially diluted 1:10, and viral plaques were counted 14 dpi by visualization of mCherry expression.

### Western blotting

Protein was isolated by scraping cells with 100 μL of Pierce RIPA lysis and extraction buffer (25 mM Tris-HCl, pH 7.6, 150 mM NaCl, 1% NP-40, 1% sodium deoxycholate, 0.1% SDS; Thermo Fisher Scientific). Lysates were incubated on ice for 30 min and sonicated 2× for 15 s to lyse cells. Lysates were quantitated by Bradford assay, and 30 µg of protein was separated with an 8% SDS-PAGE. Following separation, the protein was transferred to a nitrocellulose membrane and blocked with 5% BSA for 1 h at room temperature. The membranes were probed with anti-E1A (1:200, 8A8, Santa-Cruz), anti-E7 (1:200, ED17, Santa-Cruz), anti-actin (1:10,000, 13E5, Cell Signaling), anti-pIRF3 (1:500, 4D4G, Cell-Signaling), and anti-IRF3 (1:2000, Thermo Fisher), overnight at 4 degrees Celsius and then washed with TBS-Tween (0.1%) the following day, three times 5 min each wash. Secondary antibodies anti-mouse or rabbit-HRP (1:2000, Cell-Signaling) were probed for 1 h at room temperature and then washed an additional three times for 5 min each wash. All images were processed and imaged on Bio-Rad’s Chemidoc with chemiluminescence or by fluorescence.

### Biotin capture of nitrosylated proteins

In total, 100 µg of protein from cell lysates was subjected to a biotin-switch assay following the manufacturer’s protocol (Cayman Chemical Company). Biotinylated proteins were affinity purified with streptavidin-coated beads M-280 Dynabeads (Thermo-Fisher Scientific) overnight and washed six times with cold PBS using Life Technologies Dynamag-2. Beads were boiled in 2× Laemmli and loaded onto an 8% SDS-PAGE. The protein was separated and probed for antibodies specific to E1A or E7.

### Real-time quantitative PCR

The cells were stimulated with 10 μM of 2′3′-cGAMP, and then 24 h later, lysed with TRIZol. Samples were incubated for 5 min at room temperature, and then, 1′3′-BCP was added to each sample; in addition, the samples were shaken for 15 s. The samples were then incubated for 10 min at room temperature and then centrifuged for 12 min at 12,000 × *g*. Following centrifugation, the top aqueous layer was extracted, and then, RNA was precipitated with 100% isopropanol and incubated for 5 min at room temperature. Following incubation, the samples were then centrifuged for 10 min at 12,000 × *g*. Then, the supernatant was aspirated, and RNA pellets were washed with 75% ethanol and centrifuged for 5 min at 7,500 × *g*. Ethanol was aspirated following centrifugation, and RNA samples were dried for 10 min. The samples were then resuspended in water and incubated at 37 degrees Celsius for 5 min to increase the solubility of RNA. All sample concentrations were measured on a Thermo Fisher nanodrop.

### Statistics

All experiments in this study were analyzed using an unpaired Student *t*-test. The results were considered significant if the calculated *P*-value was <0.05, indicated by a 95% CI. All data were graphed and analyzed utilizing the program GraphPad Prism.

## Data Availability

The authors will make all data and reagents generated and described in this article available to the scientific community upon request.
